# Intraspecific Seasonal Variation of Flowering Synchronization in a Heterodichogamous Tree

**DOI:** 10.3390/plants9111509

**Published:** 2020-11-07

**Authors:** Noemi Tel-Zur, Tamar Keasar

**Affiliations:** 1 French Associates Institutes for Agriculture and Biotechnology of Drylands, J. Blaustein Institutes for Desert Research, Ben-Gurion University of the Negev, Sde-Boqer Campus, Sde Boqer 8499000, Israel; telzur@bgu.ac.il; 2 Department of Biology, University of Haifa—Oranim, Tivon 36006, Israel

**Keywords:** dichogamy, flowering rhythm, phenotypic plasticity, synchrony disruption, *Ziziphus spina-christi*

## Abstract

Heterodichogamous reproduction in plants involves two flowering morphs, reciprocal in their timing of male and female sexual functions. The degree of synchrony in floral sex phase, within and between individuals of each morph, determines the flowers’ potential fertilization partners. Complete within-morph synchrony enables across-morph mating alone, whereas unsynchronized floral sex phases may allow fertilization within a plant individual (geitonogamy) or within a morph. We documented the disruption of flowering synchrony in the heterodichogamous *Ziziphus spina-christi* towards the end of its seven-month flowering season. This desert tree has self-incompatible, protandrous, short-lived (2-day) flowers that open before dawn (‘Early’ morph) or around noon (‘Late’ morph). We counted flowers in the male and female phase on flowering branches that were sampled monthly during the 2016–2018 flowering seasons. In 2018, we also tagged flowers and followed their sex-phase distributions over two days at the start, middle, and end of the season. The switch to the female phase was delayed at the end-season (November-December), and 74% of the flowers did not develop beyond their male phase. Differences in male-phase duration resulted in asynchrony among flowers within each tree and among trees of both flowering morphs. Consequently, fertilization between trees of the same morph becomes potentially possible during the end-season. In controlled hand-pollination assays, some within-morph fertilizations set fruit. The end-season breakdown of synchronous flowering generates variability within morphs and populations. We suggest that this variability may potentially enable new mating combinations in a population and enhance its genetic diversity.

## 1. Introduction

Dichogamy entails the separation in time between the male and female functions of hermaphrodite flowers [1,2]. It has been proposed that such separation generates a two-fold selective benefit. Namely, prevention of selfing by autogamy (fertilization within the same flower), with its associated fitness costs [1,3], and reduced physical interference between the flower’s male and female sex organs [4]. In a synchronized dichogamous plant, most flowers are either in the male phase or in the female phase at any given time. Ramets of *Alstroemeria aurea* and inflorescences of *Butomus umbellatus*, for example, exhibit such synchrony [5,6]. Within-plant synchronization provides an additional advantage beyond that offered by simple dichogamy in that it reduces selfing by geitonogamy (fertilization between flowers on the same individual plant). However, perfect synchrony across an entire population would generate single-sex populations, thereby precluding any fertilization whatsoever [7,8]. Hence, synchronized flowering in dichogamous plants carries reproductive benefits alongside potential risks.

A variation of dichogamy is heterodichogamy, in which a particular species has two genetically determined plant morphs exhibiting a reciprocal timing of the floral sex phases [7,9]. In some species, one morph is protandrous (male first), while the other is protogynous (female first). In others, flower development follows the same sex-phase order in both morphs (protandry in some species, protogyny in others), but occurs at different times of the day. Thus, in heterodichogamous species, perfect synchrony in the flower development across a population is manifested as the exact concurrency of the male phase of one morph with the female phase of the other. Heterodichogamy occurs both in species with unisexual flowers and in species with bisexual flowers. The change in sex phase occurs either daily or just once during the flowering season [7]. In most studied cases, the ratio of the two morphs in natural populations is 1:1 [9]. Therefore, each individual in a population could potentially breed with any individual of the opposite morph, i.e., with ~50% of the population. The mating-opportunity cost of population-level flowering synchrony is thus reduced in heterodichogamous plants. Synchrony in flower development is indeed common in heterodichogamous species, including *Thymelaea hirsuta* [10], *Platycarya strobilacea* [11], *Machilus thunbergii* [12], *Grayia brandegei* [13], and several species of the *Juglans* genus [8,14] and of the *Acer* genus [15,16].

Even though flower development is better synchronized than would be expected at random, it is seldom perfectly coordinated within and across individuals of heterodichogamous species. For example, in *J. mandshurica*, it was found that the male and female floral phases of do not overlap within trees [8]. Yet, a small fraction (<10%) of fertilizations do occur within each flowering morph, suggesting incomplete synchrony among trees [17]. Similar low frequencies of within-tree and within-morph fertilizations, resulting from asynchronized flowering, have also been described in *J. ailanthifolia* [18], *G. brandegei* [13], and *Acer mono* [16]. Evolutionarily, incomplete synchrony may reflect a balance between opposing selection pressures: Avoidance of selfing (favoring perfectly synchronized flowering), on the one hand, and reducing pollen/mating partner limitation (favoring asynchrony), on the other hand.

The factors that regulate flowering synchrony in heterodichogamy have not been systematically studied, but seem to involve some environmental control. For instance, disruption of synchrony was observed at low temperatures in the heterodichogamous species *Acer saccharum* [15] and *Ziziphus spina-christi* [19,20]. The effect of environmental stress was also evident in a study of two natural populations of *J. ailanthifolia,* in which most individuals were dichogamous with a synchronized timing of sex-phase change. Yet some trees, which were typically small, grew in shaded habitats, or were damaged by snowfall, transiently produced flowers that did not complete a sex-phase switch [18]. Similarly, in *P. strobilacea*, some protandrous trees displayed only a male function in some years [11]. In these last two examples, some individuals of *J. ailanthifolia* and *P. strobilacea* functioned as males only, while others of the same morph concurrently completed the shift to the female phase and functioned as females. Thus, sex expression became asynchronized between trees under some conditions. Another example of environmentally induced asynchronized flowering was reported for *M. thunbergii*, in which the female phase of the flowers was delayed on rainy days, potentially leading to overlap with male-phase flowers on the same trees [12]. Such anecdotal observations suggest that some ambient conditions (such as low temperatures) interfere with synchronized flower development. They also raise a number of open questions relating to the breakdown of synchrony in heterodichogamous plants: Are both flowering morphs and sex phases equally disrupted? Is flowering synchrony disrupted to the same extent within and among plant individuals? In addition, can the disruption of synchrony allow fertilization within each flowering morph?

We addressed these questions in the heterodichogamous desert tree *Z. spina-christi* (Rhamnaceae), one of the *Ziziphus* species exhibiting heterodichogamy [19,21,22]. The self-incompatible, protandrous, short-lived (2-day) flowers of *Z. spina-christi* are pollinated mainly by flies and honeybees [23]. The flowers open before dawn in the ‘Early’ flowering morph and around noon in the ‘Late’ morph [20]. Previous studies mentioned that flowering development is highly synchronized during most of the plant’s seven-month flowering season, but that synchrony breaks down towards the end of flowering [19,20]. Here, we predicted that: (i) Flowering synchrony would be disrupted similarly in all plants of our study population, regardless of flowering morph (‘Early’ or ‘Late’) and of sex phase, since all plants share similar environmental conditions; (ii) there would be a smaller disruption of synchrony within each plant (as all flowers belong to the same genotype) than among plants; and (iii) the disruption of between-plant synchrony would generate possibilities for within-morph reproduction.

## 2. Results

**Figure 1:**
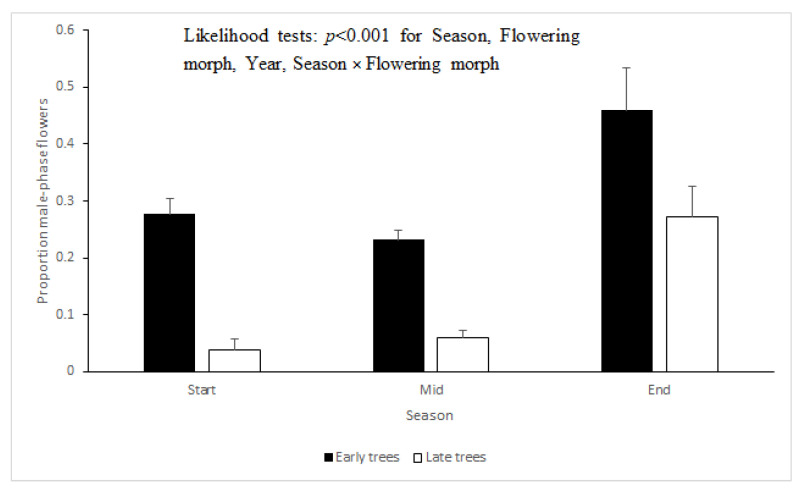
Mean per-tree proportions of male-phase flowers, in the ‘Early’ and ‘Late’ flowering morphs along the flowering season. Error bars are 1 SE. All trees were sampled between 08:30–10:30 h.

*Seasonal phenology*—During the morning hours, ‘Early’ trees carry both newly opened male-phase flowers and older flowers that had opened on the previous day and had since then transitioned into the female phase. During the morning hours, ‘Late’ trees (in which new flowers open daily around noon) display only flowers that had opened on the previous day. These flowers had been open for ~20 h and thus, had ample time to transition from the male phase to the female phase. Accordingly, the proportion of male-phase flowers was consistently and significantly higher in the ‘Early’ trees than in the ‘Late’ trees (Figure 1, generalized linear model [GLM] with Chi-square likelihood tests: *p* < 0.001 for the effect of flowering morph). The proportion of male-phase flowers was higher during the end of the flowering season than during the start or middle of the season (*p* < 0.001 for the effect of season). The interactive effect of season and morph, and the effect of the particular year, were also significant (*p* < 0.001 for both). The proportions of male-phase flowers in the population did not correlate significantly with the monthly averages of maximal daily temperatures (Spearman’s correlation coefficient: −0.374, *p* = 0.154, *n* = 16). They were, however, significantly negatively correlated with the monthly average daylight duration (Spearman’s correlation coefficient: −0.628, *p* = 0.009, *n* = 16).

The increased proportions of male-phase flowers during the end of the flowering season could potentially be explained by higher rates of flower differentiation toward the end of blooming, resulting in overall higher numbers of flowers per branch. However, there were no consistent differences between flowering morphs, seasons, and year in the number of flowers per branch. The mean ± se number of flowers per branch ranged from 5.8 ± 0.9 (in ‘Early’ trees during the end-season of 2016) to 9.7 ± 0.7 (in ‘Early’ trees during the mid-season of 2018), and the above hypothesis was thus rejected.

**Figure 2:**
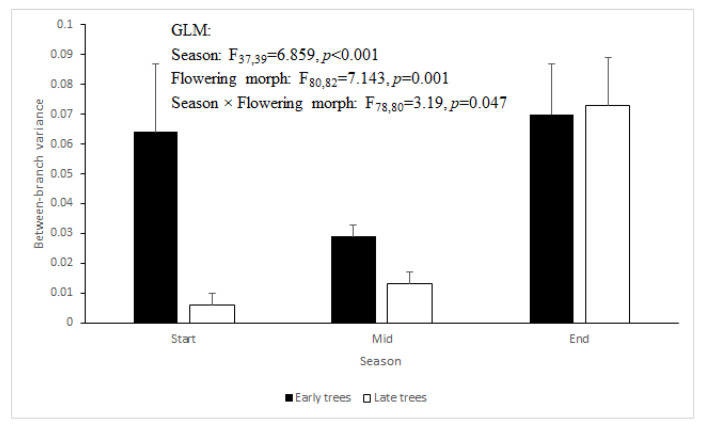
Among-branch variance of the proportion of male-phase flowers in the ‘Early’ and ‘Late’ flowering morphs along the flowering season. Means ± SE of the per-tree variances are plotted.

We used the variance in the proportion of male-phase flowers among the five branches sampled per tree as a measure of within-tree asynchrony. In perfectly synchronized trees, all five branches would have an identical proportion of male-phase flowers at any point in time, and hence, the variance would be 0. If flower development were to be less well synchronized across branches, the variance would increase. For example, if two of the branches carry only male-phase flowers, while the remaining three branches carry only female-phase flowers, then the male-phase proportions are {1, 1, 0, 0, 0} and the variance is 0.3. The between-branch variance in our samples of *Z. spina-christi* was more marked in ‘Early’ trees than in ‘Late’ trees. It reached its highest level, in the two morphs combined, during the end-season (Figure 2; GLM: F_37,39_ =6.859, *p* < 0.001 for season, F_80,82_ = 7.143, *p* = 0.001 for flowering morph, F_78,80_ = 3.19, *p* = 0.047 for season × flowering morph, the year was non-significant).

**Figure 3:**
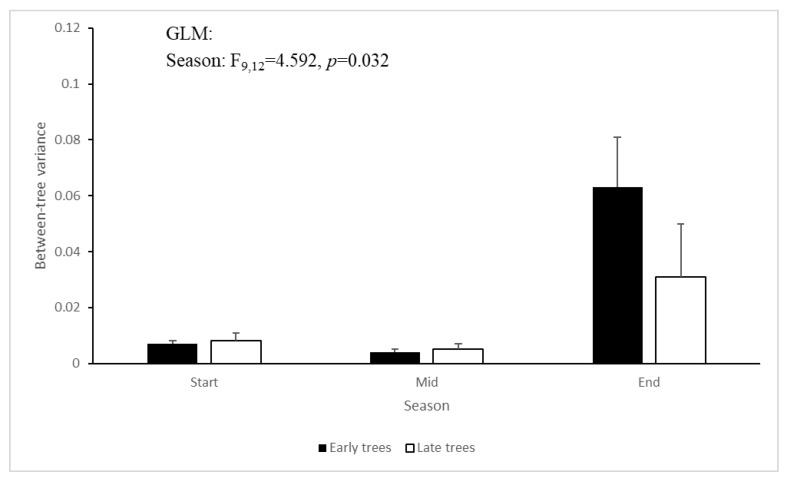
Among-tree variance of the proportion of male-phase flowers in the ‘Early’ and ‘Late’ flowering morphs along the flowering season. Error bars indicate the between-year standard errors.

Next, we used the variance in the proportion of male-phase flowers across trees as an estimator of between-tree asynchrony. For this, we first pooled the data from the five branches sampled from each tree, and calculated the per-tree proportion of male-phase flowers. We then calculated the variance of these proportions for each season-year combination separately for ‘Early’ and ‘Late’ trees. The greater the differences in the flowers’ sex-phase distribution across trees (= asynchrony), the higher the variance. The between-tree variance in the proportion of male-phase flowers was affected by season (F_9,12_ = 4.592, *p* = 0.032), also being highest at the end-season (Figure 3). The year and flowering morph did not significantly affect the asynchrony among trees.

**Figure 4:**
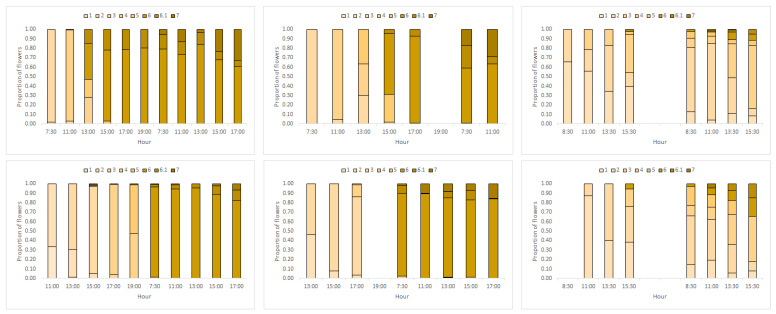
Detailed daily flower progression in ‘Early’ and ‘Late’ trees at early-, mid-, and end-season. Flower stages are described in Table 1. Darker shades correspond to more advanced flower stages.

**Figure 5:**
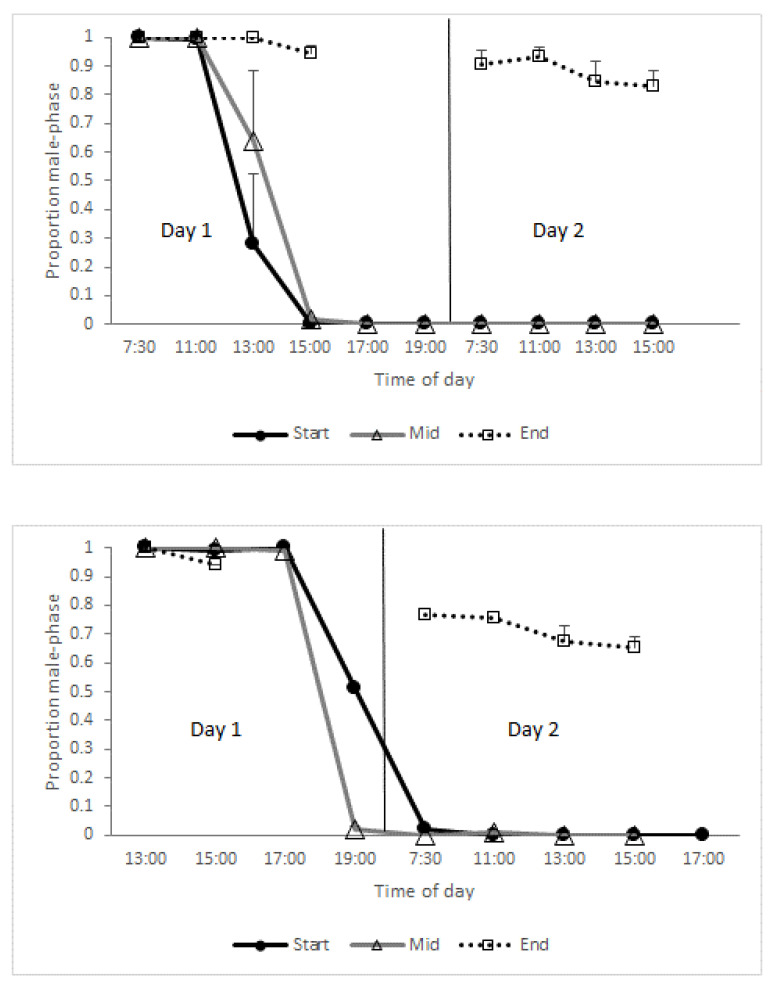
Mean per-tree proportions of male-phase flowers in the ‘Early’ (top) and ‘Late’ (bottom) flowering morphs along two consecutive days. Three trees of each flowering morph were monitored, at the start, middle, and end of the flowering season. Error bars are 1 se.

*Daily flower progression*—A detailed description of the flower phases, and their progression along the flowers’ two-day life, is given in Table 1 and Figure 4. Based on these data, we plotted the mean proportions of male-phase flowers at different times of day for ‘Early’ and ‘Late’ trees separately (Figure 5). Virtually all flowers of the ‘Early’ morph transitioned from the male to the female phase during the noon hours of their first day in the start-season and mid-season (Figure 5, top). Accordingly, all flowers were in the male phase immediately after opening (Figure 5, leftmost data points). As they progressed into the female phase, the proportions of male-phase flowers gradually declined and reached 0 by 15:00 h. In contrast, during the end-season, the greater proportion of flowers remained in the male phase for the entire two days: The average per-tree proportion of male-phase flowers at the end of the season was 0.83. Trees of the ‘Late’ morph showed a similar pattern (Figure 5, bottom). However, ‘Late’ morph trees had a significantly (*p* < 0.001) lower average proportion (0.66) of flowers that did not transition into the female phase than the ‘Early’ morph. The longer duration of the male phase, especially in the ‘Early’ morph, explains the higher proportion of male-phase flowers during the end-season (Figure 1). Note that male-phase flowers in the ‘Early’ morph were much more common in the daily flowering progression monitoring (1.0 during the morning, Figure 5) than in the seasonal phenology monitoring (<0.5, Figure 1). This is because day-2 flowers, which had reached their female phase, were included in the seasonal phenology counts but not in the monitoring of daily flower progression.

*Hand-pollination assays*—The fruit set of flowers that were hand-pollinated with the same-morph pollen was 4% (3/74), indicating some within-morph reproductive compatibility. Eight out of the 39 open-pollinated flowers from the same trees (20.5%), which served as controls, set fruit. The percent fruit set of the open-pollinated flowers was significantly higher than in the hand-pollination treatment (Fisher’s exact test, *p* = 0.008).

## 3. Discussion

Our observations confirm and quantify previous reports on the breakdown of flowering synchrony in *Z. spina-christi* towards the end of its flowering season. Our study contributes three novel insights regarding this phenomenon. First, we found that synchrony disruption occurred both within and between individual trees (Figure 2 and Figure 3). The within-tree asynchrony was manifested as varying proportions of male-phase flowers on different branches of the same tree, and varying proportions of male-phase flowers among trees indicated between-tree asynchrony. Second, we also recorded, for the first time, high within-tree asynchrony in ‘Early’ trees at the start of the season. Finally, we found that the synchrony breakdown was due to delayed or even missing transition from the male to the female phase of the flowers (Figure 4 and Figure 5). Since each flower spends the greater part of its lifetime in the male phase, at the expense of the female phase, the proportion of male-phase flowers in the population increases towards winter (Figure 1). A similar plasticity in the sexual phase duration, involving both genetic and environmental effects, has been reported for several dichogamous species (e.g., [6,24]). The frequencies of male-phase flowers in our samples were negatively correlated with daylight duration but not with the mean maximal temperature. Light, a common *Zeitgeber* of circadian phenomena, could therefore act as a physiological signal that controls the flowers’ sex-phase transition. However, other environmental cues that change seasonally (such as minimal temperature, radiation intensity, air humidity, or rainfall) may also potentially regulate floral development. To understand what governs the sex-phase transition in *Z. spina-christi*, experiments in environmentally controlled growth chambers are needed.

Our initial predictions were only partially supported by field observations. In contradiction of our first prediction, ‘Early’ trees showed higher within-tree asynchrony (i.e., more variation in the proportions of male-phase flowers, Figure 2) and lower rates of sex-phase transition (Figure 5) than ‘Late’ trees. Contrary to our second prediction of higher asynchrony between trees than within trees, the variance that we found in the proportions of male-phase flowers between branches (Figure 2) was similar to that between tree individuals (Figure 3). In contrast to the above dissonance between predictions and findings, the success of some fertilizations between same-morph trees supports our last premise that asynchronized flowering could diversify the plants’ reproductive options through within-morph mating.

**Figure A1:**
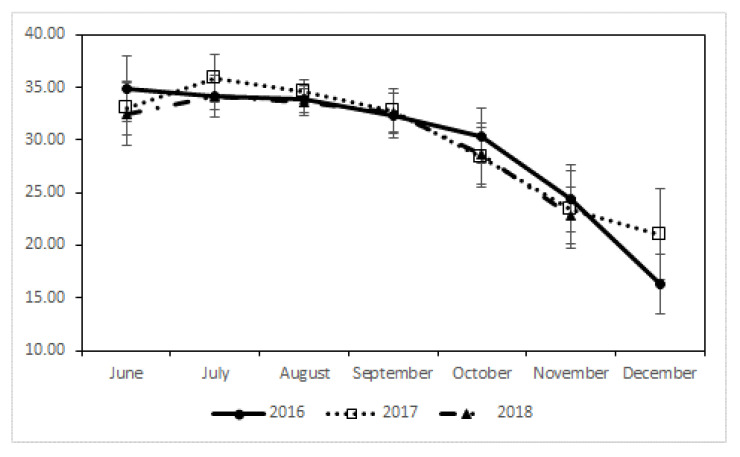
Monthly means (± SD) of the maximal daily temperatures at the field site over the 2016–2018 flowering seasons.

During the end-season, an average of 74% of flowers per tree remained in the male phase throughout the two days of monitoring. Physiologically, this may be due to resource depletion at the end of flowering, so that plants that are unable to mature fruit do not develop pistils (functional andromonoecy; [25,26]). Alternatively, the lower end-season temperatures (Appendix A Figure A1) may inhibit pistil elongation. From a reproductive point of view, these functional-male flowers can potentially fertilize female-phase flowers of the complementary morph as well as those within their morph. The hand-pollination assay showed that some of these fertilizations indeed resulted in fruiting. Thus, the disruption in synchrony may increase the trees’ pool of reproductive partners, since within-morph fertilizations can occur. The present results provide only a qualitative confirmation of the feasibility of within-morph fertilization in *Z. spina-christi*. Further work is needed to assess the frequency, timing, and impact of within-morph fertilizations in natural populations.

In our small-scale hand-pollination assay, hand-pollinated flowers set fruit at a significantly lower rate than open-pollinated control flowers. This could be caused by a partial reproductive barrier between trees of the same morph, but also possibly by physical damage to the flowers during hand pollination. Additional assays with numerous parental genotypes and with an additional control treatment of hand-pollinated between-morph flowers are needed to test these interpretations. Such a follow-up study would allow quantitative comparisons of the success of within-morph vs. between-morph fertilizations. Additional future work should also develop genetic markers to estimate the proportion of naturally developing fruit resulting from within-morph fertilizations.

The stalled development of some male-phase flowers probably also increased the within-plant transfer of pollen, via pollinating insects that moved between male-phase and female-phase flowers within each tree. This might have reduced the trees’ reproductive success through pollen waste and clogging of receptive stigmas with self-pollen. Selfing is probably prevented by *Z. spina-christi*’s self-incompatibility mechanism. It has been proposed that self-incompatibility and flowering synchrony overlap in function when they co-occur. Such redundancy may relax the selective pressure for flowering synchrony in a plant that is fully self-incompatible [27]. The breakdown of synchrony in *Z. spina-christi* during the end-season is consistent with this hypothesis.

The asynchrony in flower progression among branches of the same tree, and even among flowers on the same branch, suggests some control over development at the level of each individual flower. This notion is in agreement with molecular evidence suggesting that each and every plant cell has its own circadian clock [28]. The circadian clock in plants can be entrained by multiple environmental signals, including light, temperature, and nutrient status. While circadian-clock pathways control flower opening and heliotropism in some species [29,30], it is not yet clear whether synchronized sex-phase shifts in plants also involve the circadian system.

In a previous study, we found that flies, ancestral pollinators of *Z. spina-christi*, visit the flowers primarily during the morning hours. This biases the ‘Early’ trees (which are male-phase in the morning) towards the male function, while the ‘Late’ morph tends toward the female function and produces more fruit ([23], see also [12] for a similar specialization in *M. thunbergii*). In the present study, we found a further specialization of the ‘Early’ morph towards the male function, since more of its flowers remained in male-phase during the end-season than in the ‘Late’ morph (Figure 4). Similarly, in the heterodichogamous *T. hirsuta*, the frequency of male-phase flowers increases toward the end of the flowering season [10].

In summary, the seasonal disruption of flowering synchrony in *Z. spina-christi* (a) generates possibilities for fertilization within flowering morphs, which could enable novel mating combinations; (b) increases the flowers’ time window for pollen export (male phase), while possibly reducing the time available for pollen import (female phase); and (c) enhances the specialization of the ‘Early’ and ‘Late’ flowering morphs as pollen donors and recipients, respectively. Synchronized flowering may, thus, play a more complex role than previously appreciated in regulating the reproductive system of *Ziziphus*.

## 4. Materials and Methods

### 4.1. The Study Population

**Figure A2:**
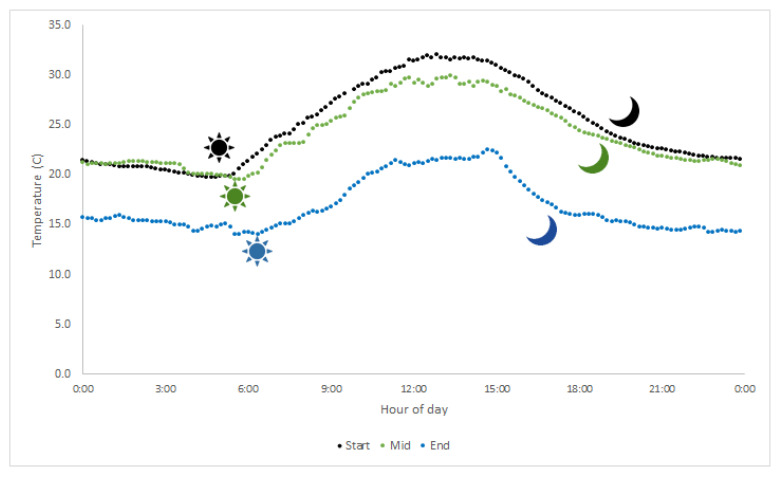
Mean temperature records, collected at 10-min intervals, for the dates on which flowering progression was monitored in 2018. The sun and moon symbols indicate the times of sunrise and sunset on the monitoring days.

**Figure A3:**
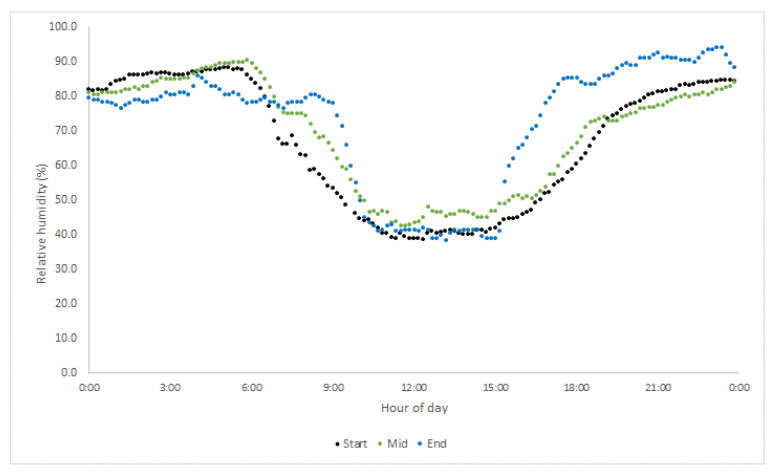
Mean relative humidity records, collected at 10-min intervals, for the dates on which flowering progression was monitored in 2018.

The flowering phenology of *Z. spina-christi* was monitored in a natural population in southern Israel (31°30′79″ N, 34°90′16″ E, elevation 350 m) during the flowering seasons of the years 2016–2018. The trees in the population were 2–7 m tall, and their ages are unknown. The study site has an average yearly temperature range of 12.4–28.1 °C, and an average annual rainfall of 213 mm. Local weather conditions for the periods of field sampling were obtained from the nearest meteorological station (see Appendix A Figure A1, Figure A2 and Figure A3). Hand-pollination assays were conducted in a planted and irrigated *Ziziphus* grove, containing multiple genotypes, at an experimental desert site (30°85′34″ N, 34°78′33″ E, elevation: 480 m, temperature range: 9.9–26.2 °C, average annual rainfall: 93 mm). They were performed in August and September (mid-season) of 2019.

### 4.2. Seasonal Flowering Phenology

Eight ‘Early’ and eight ‘Late’ trees were selected for monthly monitoring of flowering phenology. Five flowering branches (~20 cm length), facing different directions, were clipped from each tree at a height of about 1.5 m from the ground. These samples were taken once a month, during the morning hours (08:30–10:30), throughout the 2016–2018 flowering seasons. Male- and female-phase flowers were counted under a dissecting microscope, and the proportion of male-phase flowers per branch was calculated. About 8% of all flowers had folded-back anthers that contained no pollen, but their stigmata remained undeveloped (described as phase 6.1 in Table 1). Such flowers were previously classified as unisexual male flowers [19]. However, aniline blue staining indicated that pollen had germinated in the styles of about 30% of such flowers under field conditions (Tel-Zur, unpublished data), and we therefore, classified them as female flowers.

### 4.3. Monitoring of the Daily Progression of Flower Stages

This monitoring involved a separate set of observations in July (start of season), September (mid-season), and November (end of season) of 2018. We tagged about 10 branches that together carried > 50 large buds (expected to open within the next day) on each of three ‘Early’ and three ‘Late’ trees. The selected branches were oriented in different directions in all trees and on all sampling dates to account for variations in the flowers’ exposure to sunlight. ‘Early’ trees were tagged in the afternoon before the monitoring day, and ‘Late’ trees were tagged on the morning of the monitoring day. Developing fruits, open flowers, and smaller flower buds were removed from the tagged branches. Starting on the next morning for ‘Early’ trees or at noon for ‘Late’ trees, we recorded the numbers of male- and female-phase flowers on the tagged branches at 3-h intervals until sunset for two consecutive days.

### 4.4. Pollination Procedure

Asynchronized flowering in the late season may enable viable pollen from a male-phase flower to reach a female-phase flower of the same morph, but can such pollen transfer actually lead to fertilization and fruiting? To address this question, we performed controlled hand pollinations between two genotypes of the same morph as follows: A day before anthesis, 74 flower buds from two trees—one ‘Early’ and one ‘Late’—were selected to be used as female parents. These buds were tagged and covered with paper bags to prevent uncontrolled fertilization. Other flowers, flower buds and developing fruits on the same branches were removed. To facilitate pollination with a pollen donor from the same morph, flowers were collected from three other trees during their male phase and stored at 4 °C for 4–20 h. When the bagged flowers reached the female phase (identified by a clear bilobed and elongated stigma), the surface of the receptive stigmas was covered with pollen from the ‘cold-storage’ flowers by using fine forceps. The pollinated flowers were bagged for 2 additional days to prevent contamination and then examined for fruit development 1 month later. Thirty-nine additional tagged flowers from the same trees were not manipulated in any way and served as open-pollinated controls. The fruit set of these flowers was also recorded. This control aimed to ensure that the tested trees were capable of fertilization and fruiting on the dates of the hand pollination assays.

### 4.5. Data Analysis—Seasonal Phenology

We defined June-July as ‘start-season,’ August-October as ‘mid-season’, and November-December as ‘end-season.’ To test for factors influencing the proportion of male-phase flowers, we coded the sex-phase of each flower as a binomial response variable (1—male, 0—female). The effects of year, season, flowering morph, and the season×flowering morph interaction on this variable were calculated using a GLM for binomially distributed data with a logit link function.

To estimate flowering asynchrony, we first calculated the variance in the proportions of male-phase flowers between branches of each tree in each season (*n* = 88 samples, each sample represents a particular tree-season-year combination). This calculation served as a measure of the disruption of within-tree flowering synchronization, since the variance among branches increases as synchronization declines. Next, we calculated the proportion of male-phase flowers for each tree-season-year combination, pooling the data from all branches of each tree. The variance of these proportions served as a proxy for synchrony disruption among trees. We used generalized linear models (GLMs) with a gamma error structure and a log link function to test the effects of year (2016/2017/2018), season (start/mid/end), flowering morph (‘Early’/’Late’), and the season × flowering morph interaction on the between-branch and between-tree variance.

We started all GLM analyses (for proportion of male-phase flowers and for asynchrony) by calculating the full models with all explanatory variables. To identify statistically significant effects, we compared them with simplified models, from which we gradually removed explanatory variables. We used likelihood ratio tests to compare models with binomially distributed data, and F-tests for comparing models with gamma-distributed data.

### 4.6. Daily Flower Progression

We calculated the proportion of male-phase flowers per tree at each hour of observation and averaged the proportions over the three trees of each flowering morph.

All statistical analyses were conducted in R version 3.5.1 [31].

## Figures and Tables

**Table 1:** Definition of floral stages in *Ziziphus spina-christi.*

Stage		Image (by N. Tel-Zur)	Sex-Phase
Present Study	Galil and Zeroni, 1976		
1		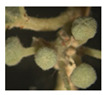	Before anthesis
2	A	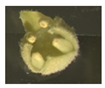	Anthesis
3	B	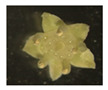	Male phase
3	B1	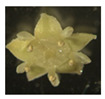	Male phase
4	C	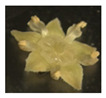	End of the male phase
5	D	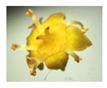	Male + female phasesGalil and Zeroni – Early female
6	E	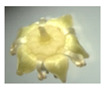	Female phase
6.1		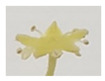	Female phase with no pistil elongation (present study), Male phase (Galil and Zeroni, 1976)
7	F	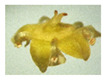	Necrotic stigma
